# Differential relationship of uric acid to mortality and clinical biomarkers of aging according to grip strength in older adults: a cohort study

**DOI:** 10.18632/aging.202820

**Published:** 2021-04-04

**Authors:** Sin-Mei Guo, Yen-Tze Liu, Sin-Ru He, Ming-Shiang Wu, Wei-Ting Tseng, Ray-Chin Wu, I-Chien Wu

**Affiliations:** 1 The Department of Family Medicine, Changhua Christian Hospital, Changhua, Taiwan; 2 Institute of Population Health Sciences, National Health Research Institutes, Zhunan, Taiwan; 3 Institute of Medicine, Chung Shan Medical University, Taichung, Taiwan; 4 Department of Holistic Wellness, MingDao University, Changhua, Taiwan; 5 Graduate Institute of Biomedical Sciences, China Medical University, Taichung, Taiwan

**Keywords:** biomarkers, oxidative stress, epidemiology, physiology, aging

## Abstract

Uric acid is both a pro-oxidant and antioxidant. We investigated serum uric acid's association with mortality and aging biomarkers in older adults with varying levels of grip strength. A total of 5329 community-dwelling adults aged ≥55 years underwent assessments of serum uric acid levels, grip strength, and biomarkers of diverse physiological systems. The primary outcome was all-cause mortality. We observed a significant (P < .001) interaction between uric acid levels and grip strength on all-cause mortality risk. Among participants with low grip strength, a nonlinear association (P for nonlinearity = .006) was observed between serum uric acid levels and mortality risk after multivariate adjustment. Compared with participants with neither extreme uric acid levels nor low grip strength, those with a combination of high serum uric acid and low grip strength exhibited greater risks of mortality (adjusted hazard ratio [aHR], 1.52; 95% confidence interval [CI], 1.15–2.02) and deviations in biomarkers of specific systems, so did those with a combination of low serum uric acid and low grip strength (aHR, 1.52; 95% CI, 1.13–2.05). In conclusion, there was a J-shaped association between serum uric acid and the risk of all-cause mortality in older adults. This was primarily true for those with low grip strength.

## INTRODUCTION

Aging is accompanied by increasing levels of reactive oxygen species; when they exceed a threshold, they can be detrimental and accelerate biological aging [1–6]. Although uric acid is widely known as a proinflammatory waste product of the metabolic breakdown of purine nucleotides with deleterious effects, emerging experimental results suggested that it could be a double-edged sword with respect to modulation of oxidative stress. It is a pro-oxidant in hydrophobic environments and promotes intracellular accumulation of reactive oxygen species, macromolecule damage, and cell/tissue dysfunction [7–9]. On the other hand, uric acid can also act as a strong antioxidant capable of removing free radicals in hydrophilic environments [10–12]. Thus, in theory, high or low serum uric acid levels could exaggerate the oxidative stress in aging, thereby accelerating aging in diverse physiological systems and increasing the risk of death.

Previous studies indicated the association of high serum uric acid levels with the increased risks of multiple adverse health outcomes [13–16]. Despite these observations, little is known about the relationship between serum uric acid levels and death risk during aging. Grip strength can predict a healthy lifespan [17–20]. Low grip strength is associated with an increased risk of premature death and a lower likelihood of healthy aging. Grip strength has been increasingly applied as a clinical measure of aging to capture heterogeneity in the degree of biological aging, including oxidative stress in aging [17, 18, 21–23].

Unlike fat, skeletal muscle is a water-rich tissue that holds a large proportion of water in the human body [24]. It is well-recognized that the composition of the human body changes during aging [25]. Whereas fat mass increases, muscle mass decreases with increasing age, and so does the muscle function (strength). In addition, aging is accompanied by an increased susceptibility to dehydration and water loss in the body [26]. As such, there is usually a loss of water (cellular hydration) in skeletal muscle during aging, which may also be associated with muscle strength loss [27]. Altogether, these changes result in a contraction in the hydrophilic environment's volume in the body and skeletal muscle, which in turn may set the stage for increased oxidative stress induced by low uric acid levels. Also, the resultant hydrophobic environment could favor increased oxidative stress caused by high uric acid levels.

This study's primary aim was to investigate the relationship between serum uric acid levels and mortality risk across a spectrum of grip strengths. We hypothesized that high and low serum uric acid levels are associated with increased risks of all-cause mortality, particularly in older adults with low grip strength. We also hypothesized that older adults with both low grip strength and high or low serum uric acid levels would display marked deviations in the levels of clinical biomarkers, reflecting age-related changes in physiological systems.

## RESULTS

### Participants' characteristics

The baseline characteristics of the study participants are presented in Table 1 and Supplementary Table 1. The mean age of the participants was 69.4 ± 8.2 years, and 52% were women. The mean serum uric acid levels were 6.0 mg/dL (range, 0.5–14.2). A total of 2732 participants had low grip strength, whereas 2597 participants had high grip strength. Of the 5329 participants, 1070 (19.5%) died over a mean follow-up period of 5.81 years (range, 0.14–11.23).

**Table 1:** Comparisons of baseline characteristics between study participants with low and high grip strength.

**Characteristics**	**Grip strength^a^**			***P*^b^**
	**All** **(*n* = 5329)**	**Low** **(*n* = 2732)**	**High** **(*n* = 2597)**	
Uric acid, mg/dL, mean (SD)	6.0 (1.6)	6.1 (1.6)	5.9 (1.5)	<.001
Age, years, mean (SD)	69.4 (8.2)	72.7 (7.9)	65.9 (6.8)	<.001
Women	2788 (52.3)	1444 (52.9)	1344 (51.8)	.420
Married	3975 (74.6)	1909 (69.9)	2066 (79.6)	<.001
Smoking				.025
Never	3757 (70.5)	1904 (69.7)	1853 (71.4)	
Ever	886 (16.6)	490 (17.9)	396 (15.2)	
Current	686 (12.9)	338 (12.4)	348 (13.4)	
Alcohol drinking				<.001
Never	3219 (60.4)	1702 (62.3)	1517 (58.4)	
Former drinker	557 (10.5)	345 (12.6)	212 (8.2)	
Current drinker	1553 (29.1)	685 (25.1)	868 (33.4)	
Betel nut chewing				<.001
Never	4670 (87.6)	2424 (88.7)	2246 (86.5)	
Former chewer	491 (9.2)	248 (9.1)	243 (9.4)	
Current chewer	168 (3.2)	60 (2.2)	108 (4.2)	
Body mass index				<.001
Underweight	152 (2.9)	115 (4.2)	37 (1.4)	
Normal	1595 (29.9)	880 (32.3)	715 (27.5)	
Overweight	2600 (48.8)	1288 (47.2)	1312 (50.5)	
Obese	976 (18.3)	443 (16.3)	553 (20.5)	
Hypertension	2860 (53.7)	1617 (59.2)	1243 (47.9)	<.001
Diabetes mellitus	1523 (28.6)	879 (32.2)	644 (24.8)	<.001
Cardiovascular disease	1144 (21.5)	688 (25.2)	456 (17.6)	<.001
Stroke	285 (5.3)	211 (7.7)	74 (2.8)	<.001
Lung disease	174 (3.3)	109 (4.0)	65 (2.5)	.002
Cancer	305 (5.7)	172 (6.3)	133 (5.1)	.065
Arthritis	934 (17.5)	535 (19.6)	399 (15.4)	<.001
Chronic kidney disease	863 (16.2)	610 (22.3)	253 (9.7)	<.001
Uric acid lowering drug use	156 (2.9)	99 (3.6)	57 (2.2)	.002
Years of observation, years, mean (SD)	8.7 (2.2)	8.3 (2.5)	9.2 (1.7)	<.001

Note: Data are presented as *n* (%) unless otherwise specified.

^a^Low grip strength: women ≤22 kg, men ≤36 kg.

^b^Continuous variables were analyzed using one-way ANOVA, whereas categorical variables (proportions) were analyzed using the chi-square test.

### The relationship between serum uric acid levels and mortality risk differed by grip strengths

In the Cox proportional-hazards regression models, a significant effect was observed for the interaction between serum uric acid levels and grip strength on the risk of all-cause mortality after adjustment (*P* for interaction < .001 when grip strength was analyzed as a categorical variable; *P* for interaction < .001 when grip strength was analyzed as a continuous variable). We conducted subsequent analysis stratified by grip strength.

**Figure 1:**
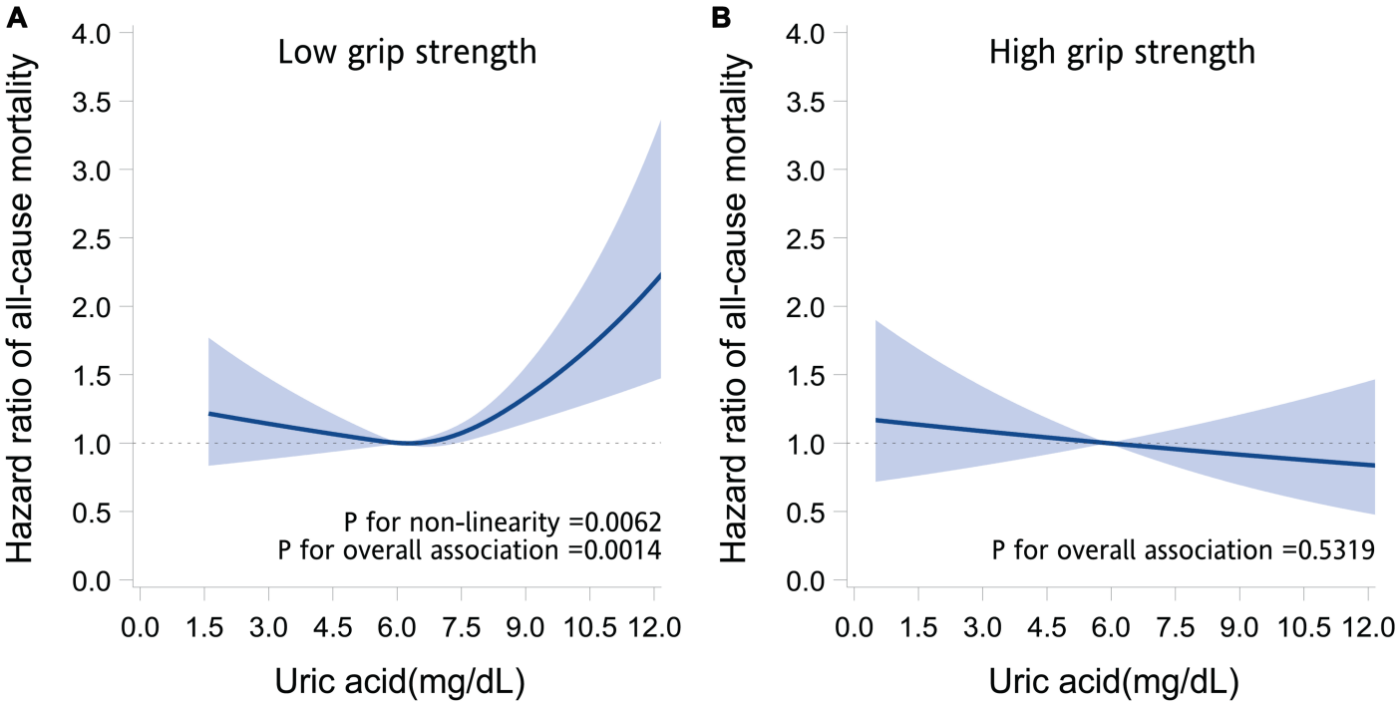
**Association between serum uric acid and the risk of death.** The graphs present the adjusted hazard ratios of all-cause mortality for serum uric acid levels in older adults with low grip strength (**A**) and high grip strength (**B**). The hazard ratios (solid line) and 95% confidence intervals (band) were estimated by fitting restricted cubic spline Cox regression models, in which uric acid was modeled as a continuous variable with splines having 4 knots placed at the 5th, 35th, 65th, and 95th percentiles. Models were adjusted for age, sex, marital status, behavioral characteristics, body mass index, chronic diseases, and use of uric acid–lowering drugs.

Among participants with low grip strength, restricted cubic spline regression analyses revealed a nonlinear J-shaped association (*P* for nonlinearity = .006; *P* for overall association = .001) between serum uric acid levels and mortality risk after adjustment (Figure 1A). Both high and low serum uric acid levels were associated with increased risks of mortality. The lowest risk was at serum uric acid levels in the range of 5.2–5.9 mg/dl. As compared with participants with serum uric acid levels in the range of 5.2–5.9 mg/dl (the reference group), those with higher or lower uric acid levels tended to have higher mortality risks (Supplementary Figure 1). By contrast, among participants with high grip strength, the serum uric acid level was not significantly associated with mortality risk after adjustment (*P* for overall association = .53; Figure 1B).

**Figure 2:**
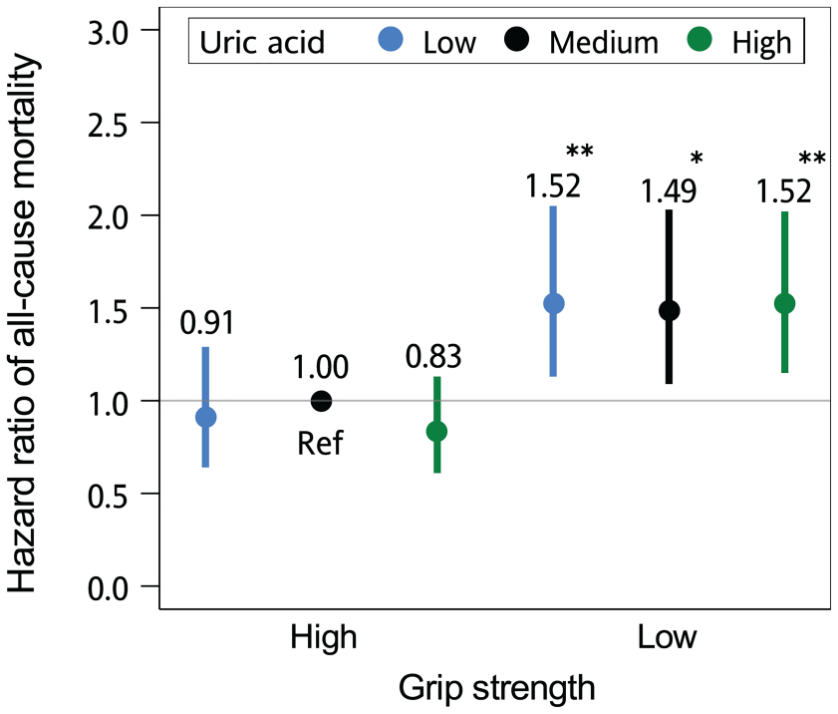
**The joint effect of grip strength and serum uric acid level on the risk of death.** The hazard ratios of all-cause mortality according to grip strength (high [>22 kg in women; >36 kg in men] and low [≤22 kg in women; ≤36 kg in men]) and serum uric acid levels (low [<5.2 mg/dl], medium [5.2–5.9 mg/dl] and high [≥6 mg/dL]) are presented. The multivariate models were adjusted for age, sex, marital status, behavioral characteristics, body mass index, chronic diseases, and use of uric acid–lowering drugs. Error bars indicate 95% confidence intervals. ^*^P < .05; ^**^P < .01, compared with the reference group (Ref).

The study population was divided into 6 groups based on grip strength (high and low) and uric acid (low [<5.2 mg/dl], medium [5.2–5.9 mg/dl] and high [≥6 mg/dL]). Figure 2 and Supplementary Table 2 present the adjusted hazard ratios (aHRs) for all-cause mortality among participants of each group. Participants with low (aHR, 0.91; 95% confidence interval [CI], 0.64–1.29; *P* = .595) or high (aHR, 0.83; 95% CI, 0.61–1.13; *P* = .240) serum uric acid levels but without low grip strength exhibited similar risks of mortality to those with medium levels of serum uric acid. The mortality risk was increased in those with low grip strength but without low or high serum uric acid levels (aHR, 1.49; 95% CI, 1.09–2.03; *P* = .012), and in those with both low grip strength and low (aHR, 1.52; 95% CI, 1.13–2.05; *P* <.001) or high serum uric acid levels (aHR, 1.52; 95% CI, 1.15–2.02; *P* <.001), suggesting an effect only in the low grip strength group.

In the sensitivity analysis, no qualitative change in the results was observed. A significant effect of the interaction between serum uric acid levels and grip strength was observed on the risk of all-cause mortality (*P* for interaction < .001 when grip strength was analyzed as a categorical variable; *P* for interaction < .001 when grip strength was analyzed as a continuous variable). Results of subsequent analyses stratified by grip strength are shown in Supplementary Figures 2–7. After controlling for confounders, the combination of low grip strength and low or high serum uric acid was associated with particularly increased mortality risk (Supplementary Tables 3, 4, and 5, Supplementary Figures 8, 9, and 10).

### Older adults with different serum uric acid levels and grip strengths exhibited different profiles of change in clinical biomarkers of aging

**Figure 3:**
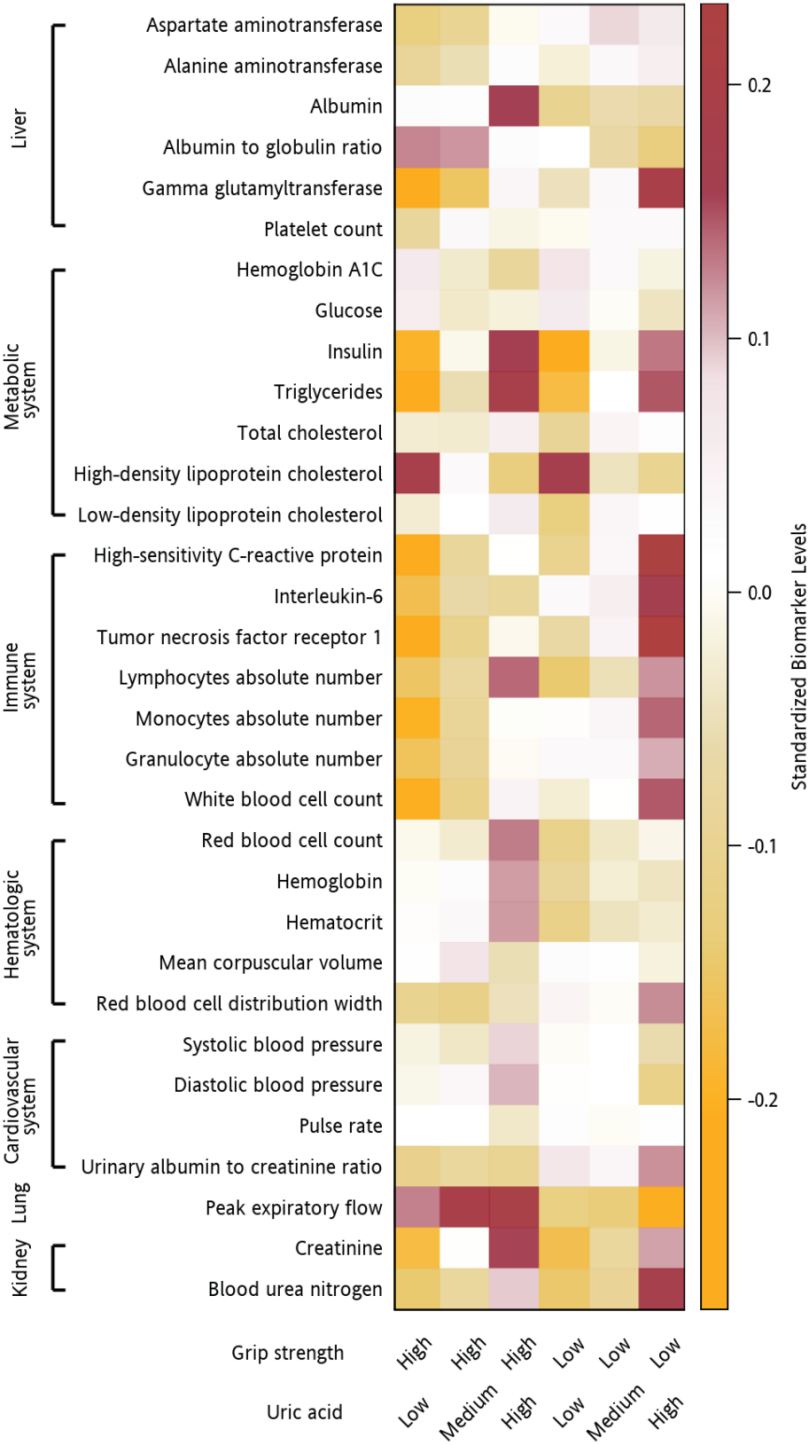
**Clinical indicators of biological aging in participants with different serum uric acid levels and grip strengths.** Heatmap of the adjusted mean levels of clinical indicators of biological aging organized by the physiological system (liver, metabolic system, cardiovascular system, lungs, kidneys, immune system, and hematologic system) in participants with different combinations of serum uric acid levels and grip strengths. The original data were standardized (non-normally distributed data were normalized first). A value of 1 indicates that the mean value for the group was one standard deviation higher than the mean value for the entire cohort. The values are represented by different colors. Brown indicates a higher mean biomarker value for that group than the mean for the entire cohort, whereas orange indicates a lower mean biomarker value for that group than the mean for the entire cohort.

To explore the accompanying changes in physiological systems, we examined the levels of 32 clinical indicators of biological aging representing diverse physiological systems (the liver, metabolic system, cardiovascular system, lungs, kidneys, immune system, and hematologic system) among older adults with different serum uric acid levels and grip strengths. Participants with different serum uric acid levels and grip strengths exhibited different change profiles (Figure 3). Participants with a combination of low grip strength and high serum uric acid exhibited deviations predominantly in immune-system biomarkers (increased hs-CRP [*P* < .001], IL-6 [*P* < .001], TNFR1 [*P* < .001], WBC count [*P* < .001], absolute lymphocyte count [*P* < .001], absolute monocyte count [*P* < .001], and absolute granulocyte count [*P* < .001]), metabolic-system biomarkers (increased insulin [*P* < .001] and triglycerides [*P* < .001] and decreased HDL cholesterol [*P* <.001]), liver-system biomarkers (increased GGT [*P* <.001], AST [*P* = .026], ALT [*P* = .041] and decreased A/G ratio [*P* <.001]), cardiovascular-system biomarkers (decreased DBP [*P* <.001] and increased ACR [*P* <.001]), kidney biomarkers (increased Cr [*P* <.001] and BUN [*P* <.001]), and lung biomarkers (decreased PEF [*P* <.001]). Those with a combination of low grip strength and low serum uric acid had similar deviations in lung biomarkers (decreased PEF [*P* <.001]) but not in the biomarkers of the immune system (decreased hs-CRP [*P* = .032] and absolute lymphocyte count [*P* = .001]), metabolic system (decreased insulin [*P* <.001], triglycerides [*P* <.001], LDL cholesterol [*P* <.001] and total cholesterol [*P* = .015] and increased HDL cholesterol [*P* <.001], HbA1C [*P* = .009], glucose [*P* = .036]), liver (decreased albumin [*P* = .008]), cardiovascular system, and kidneys (decreased Cr [*P* <.001] and BUN [*P* <.001]). Prominent deviations in hematologic system biomarkers (decreased RBC count [*P* = .003], hematocrit [*P* = .001] and hemoglobin [*P* = .005]) were particularly observed in these older adults.

**Figure 4:**
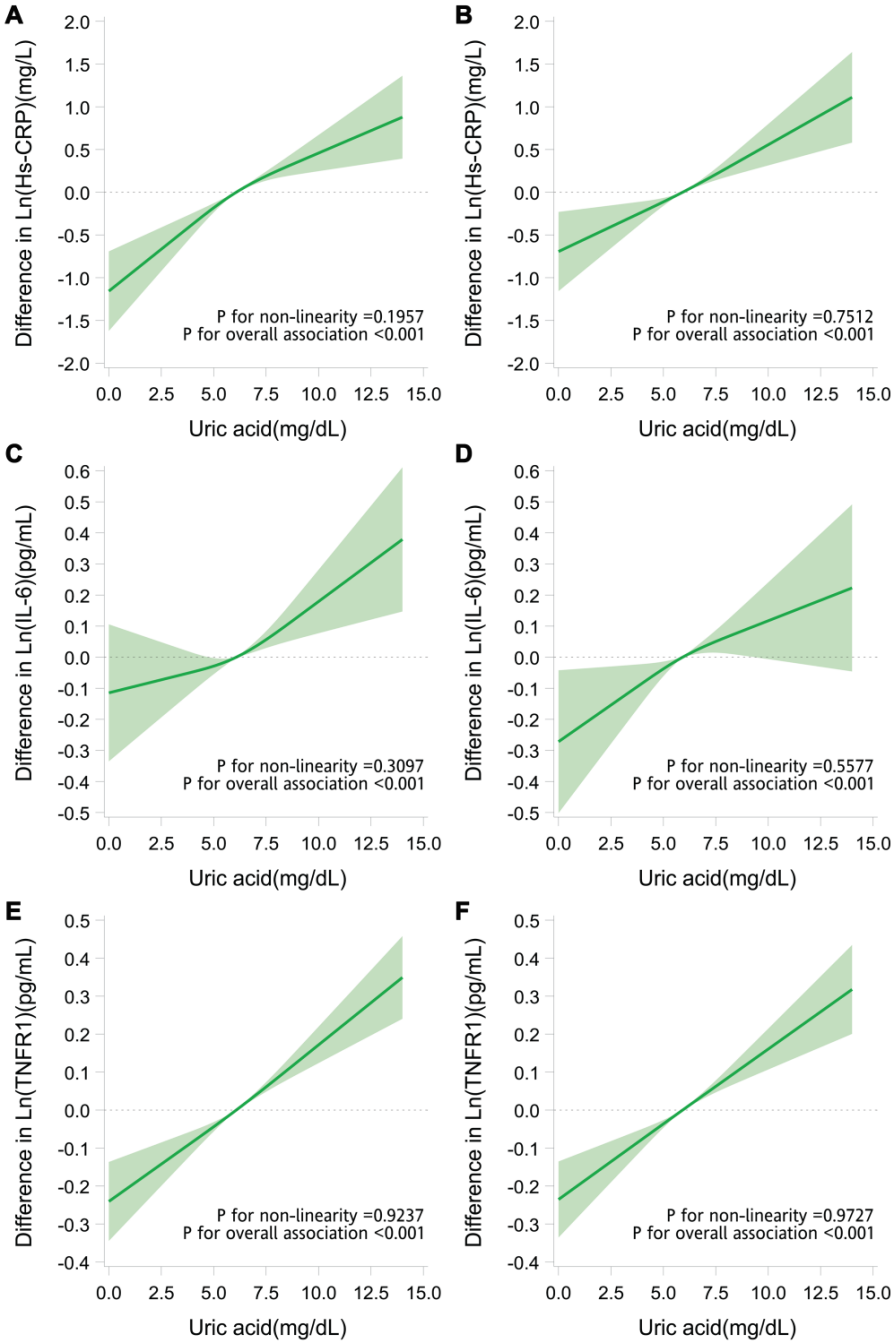
**Adjusted dose-response relationship of serum uric acid with inflammatory markers.** Difference (solid line) in levels of hs-CRP (**A**, **B**), IL-6 (**C**, **D**), and TNFR1 (**E**, **F**) associated with the uric acid levels and the mean uric acid levels (6.1 mg/dL in older adults with low grip strength and 5.9 mg/dL in those with high grip strength) in older adults with low grip strength (**A**, **C**, **E**) and in those with high grip strength (**B**, **D**, **F**). Data were obtained from restricted cubic spline regression models with the natural log-transformed inflammatory marker levels (continuous variables) as the dependent variables and uric acid level (continuous variable with splines having 3 knots placed at the 5th, 50th, and 95th percentiles) and potential confounders (age, sex, marital status, behavioral characteristics, body mass index, chronic diseases, and use of uric acid–lowering drugs) as the independent variables. The band denotes 95% confidence intervals.

Figure 4 presents the analysis results in which we regressed the levels of serum hs-CRP, IL-6, and TNFR1 against the levels of serum uric acid and potential confounders. Linear associations (*P* for nonlinearity > .05) between serum uric acid and serum hs-CRP, IL-6, and TNFR1 were observed in older individuals with low grip strength. The levels of serum uric acid were significantly associated with those of serum hs-CRP, IL-6, and TNFR1 (*P* < .001). The higher the uric acid levels were, the higher the hs-CRP, IL-6, and TNFR1 levels were.

## DISCUSSION

In this prospective cohort study, we delineated the relationship between serum uric acid level and all-cause mortality in adults aged 55 years and older with varying grip strength levels. Serum uric acid levels at either end of the spectrum were associated with increased risks of all-cause mortality. Notably, the increased mortality risks were primarily observed in older adults with low grip strength. Moreover, the combination of low grip strength and low or high serum uric acid was associated with substantial deviations in various physiological systems' biomarkers.

Previous studies showed that high uric acid levels are associated with increased risks of multiple adverse health outcomes [13–16]. However, it remains unclear how high uric acid levels are associated with an increased risk of poor health. Moreover, despite mounting evidence supporting a high serum uric acid level as a risk factor, controversies exist. For instance, which individuals (other than those with gout) can benefit from urate-lowering therapy remains unclear; moreover, when indicated, it is unclear whether urate-lowering therapy should aim to reduce serum uric acid to levels as low as possible [12, 28].

In the present study, we observed a significant effect of the interaction between serum uric acid levels and grip strength on the risk of death after adjustment for multiple potential confounders and even after considering possible reverse causation. A nonmonotonic relationship between uric acid levels and death's risk was only observed in older adults with low grip strength. This phenomenon implies that grip strength modifies the relationship between uric acid levels and the risk of death. The processes underlying loss of muscular strength might take part in the pathways linking high and low serum uric acid levels with health risks and drive the unique nature of their relationships in older adults.

Low grip strength predicts an increased risk of premature death and a lower likelihood of healthy aging. Increases in oxidative stress and inflammation play major roles in the pathogenesis of muscle mass loss and functional decline during aging [6, 21]. During aging, the production of reactive oxygen species (ROS) and reactive nitrogen species increases, mainly due to deteriorating mitochondrial dysfunction caused by numerous age-related changes (e.g., mitochondrial DNA mutations, deletions, and damage) [4, 5]. A large body of evidence indicates that high levels of oxidative stress caused by mitochondrial dysfunction directly contribute to muscular function loss during aging [3, 23].

Skeletal muscle is among the largest tissues in the body and is rich in water and electrolytes [24]. Approximately 76% of its weight is water. Water homeostasis plays a pivotal role in maintaining muscle function and systemic physiology [29, 30]. Unfortunately, advancing age is typically associated with a loss of muscle mass and muscle function, along with a volume reduction of the body’s hydrophilic environment and a concomitant volume expansion of the hydrophobic environment [31]. To make things worse, older adults are at higher risks of dehydration than younger adults, leading to further water loss and shrinkage of the hydrophilic environment in the body and skeletal muscle [26].

As described, low grip strength is typically accompanied by an expansion of a hydrophobic environment in the body. In a hydrophobic intracellular environment, uric acid could act as a pro-oxidant agent by, for instance, producing uric acid–derived radicals and increasing NADPH oxidase activity and mitochondrial oxidative stress [7–9]. Increased intracellular uric acid, as indicated by high serum uric acid levels [32], can thus worsen intracellular ROS accumulation during aging, leading to cumulative tissue damage. Moreover, these intracellular events could induce inflammation by, for instance, activating mitogen-activated protein kinases (e.g., extracellular signal-regulated kinase ½ and p38 MAPK) and downstream nuclear transcription factors (e.g., nuclear factor κB and activator protein-1) [8, 33, 34]. Moreover, inside the cells, uric acid could exert ROS-independent proinflammatory effects by directly activating mitogen-activated protein kinases and, subsequently, inflammatory signaling pathways [34]. The activated nuclear transcription factors, in turn, drive the expression and secretion of multiple inflammatory mediators, including TNF-α and IL-6 [35]. Indeed, we observed that a higher uric acid level was associated with higher levels of hs-CRP, IL-6, and TNFR1. These circulating proinflammatory molecules are known to be predictors of age-related morbidity and mortality [6]. These intracellular oxidative and proinflammatory effects of uric acid have been reported in *in vitro* experiments of endothelial cells, vascular smooth muscle cells, adipocytes, pancreatic islet cells, hepatocytes, and renal tubular cells [7, 8, 36–39], and may thus contribute to pathologic changes in multiple human organs/systems. Interestingly, in the current study, we observed marked deviations in not only aging indicators of the immune system but also in those of the cardiovascular system, metabolic system, liver, kidney, and lungs in frail older adults with high serum uric acid. Our results indicated that increased intracellular uric acid might be associated with biological aging, in addition to its better-known roles in multiple age-related diseases.

In contrast to the conclusions of previous investigations [40, 41], we found that low uric acid levels were associated with an increased risk of all-cause mortality in older adults with low grip strength. Thus, lower uric acid levels may not be better. This finding is consistent with emerging evidence indicating an inverse relationship between uric acid levels and health risk [12]. For instance, lower serum uric acid levels were associated with an increased risk of neurodegeneration and lung disease [42–44]. Another study even suggested that raising serum uric acid levels may provide protection [45]. These paradoxical associations between low serum uric levels and health risks warrant mechanistic explanation.

As described, low grip strength is typically accompanied by not only an expansion of the hydrophobic environment but also a reduction of the hydrophilic environment in the body. Unlike in the intracellular hydrophobic environment, uric acid is a potent antioxidant in hydrophilic extracellular environments [10–12, 46, 47]. In humans, approximately half of the antioxidant capacity of plasma results from uric acid's antioxidant ability. Decreasing serum uric acid levels by infusing a recombinant urate oxidase caused an increase in oxidative stress markers [10]. Increasing serum uric acid levels through direct infusion was demonstrated to alleviate oxidative stress resulting from high-intensity exercise [48]. Thus, by reducing the plasma antioxidant capacity, low levels of blood uric acid may, in theory, paradoxically increase the oxidative stress during aging systemically to a harmful level, particularly when the volume of water in the body is low. Although uric acid's pathogenic intracellular effects have been fairly well demonstrated experimentally, the potential protective extracellular effects of uric acid warrant further investigation.

We conducted regression analyses to investigate the proinflammatory actions of uric acid across its levels. With an increase in serum uric acid levels, the blood levels of inflammatory biomarkers increased. Lower serum uric acid levels were associated with lower levels of inflammatory biomarkers. These results lead to the speculation that although the increased risk of mortality in frail older adults with high uric acid levels may be related to uric acid's proinflammatory properties, the increased risk of mortality in those with low uric acid levels may not be related to inflammation. Through the examination of the levels of 32 biomarkers of diverse physiological systems that frequently change during aging, we discovered that participants with both low serum uric acid and low grip strength exhibited not only an increased risk of death but also marked deviations in the levels of biomarkers of the lungs and hematologic system, indicating impaired physiological function. It is important to note that, because of their unique physiological roles and anatomy, the erythrocytes and respiratory systems are exposed continuously to ROS's environmental sources [49, 50]. Defense against the high levels of oxidative stress is particularly essential to their physiological function, and extracellular uric acid may play a role in this [47, 50].

Serum uric acid has been proposed as a marker of nutrition, and low serum uric acid levels have been correlated with reduced consumption of purine-rich meat and seafood [51]. Studies have indicated that older malnourished people who consumed less meat and saturated fat had lower serum uric acid levels compared with community-dwelling healthy older adults and that uric acid is positively associated with grip strength, although some studies reported the opposite [52–57]. As shown in Supplementary Tables 2, 3, 4, and 5, participants with low BMI exhibited increased mortality risks. However, after adjusting for BMI, the presence of both low grip strength and low serum uric acid still conferred an increased mortality risk on the participants. Tseng et al. recently found that low uric level was associated with an increased risk of mortality in malnourished older adults [58]. Extending these findings, our results suggested that, in malnourished older adults, the presence of both low grip strength and low serum uric acid may be associated with an even greater mortality risk caused by the underlying mechanisms described above.

Due to estrogen's influence, the uric acid level is generally higher in men than in women [59]. This trend may not be applicable to aged individuals. Accumulating data indicated that the relationship between serum uric acid and death's risk does not differ in men and women [60, 61]. In line with this evidence, we did not observe significant interaction effects of sex in this study.

To our knowledge, this is the first study to investigate the relationships between uric acid, muscle strength, and mortality in older adults. This study's strengths include its moderate sample size, which allowed a complete delineation of the impact of uric acid through the construction of nonparametric nonlinear regression models. The multiple carefully assessed data on comorbidities, and other health-related characteristics of the participants allowed us to take into account the effects of potential confounding variables in the analysis. Through measurement of multiple biomarkers representing diverse physiological systems, we could conduct more in-depth investigations and obtain insights into the potential system-level mechanisms. Our study had several limitations. First, this was an observational study. The observed relationships have to be interpreted with caution because observational studies have limitations concerning the causal direction of the effects. Second, although we adjusted for multiple potentially confounding factors, residual confounders' effects could not be excluded. Uric acid-lowering drugs have been associated with increased all-cause mortality and might have caused bias in our results. To assess this, we conducted a sensitivity analysis in which participants taking these drugs were excluded. The results of the analysis were consistent, and the statistical significance remained undiminished. Third, some disorders were self-reported; thus, information bias could not be excluded. Finally, the participants were community-dwelling individuals. Therefore, the observations and conclusions might not be generalizable to older adults with severe illnesses and disabilities.

In summary, the present data provided evidence for the differential relationship of serum uric acid level with the risk of death from all causes in older adults. We demonstrated a nonlinear (J-shaped) association between serum uric acid and mortality risk, which varied with grip strength. The lower the grip strength, the stronger the association. Significant deviations in the biomarkers of physiological systems that frequently change during aging were observed in participants with high or low serum uric acid levels and low grip strength. These results implied that, in clinical practice, simply measuring grip strength may aid in identifying older adults with either high or low serum uric acid level who are at a particularly increased risk of adverse health outcomes. Future research, including randomized controlled trials, is warranted. Moreover, whether therapies that achieve uric acid homeostasis yield health benefits in these high-risk older adults remains to be established.

## MATERIALS AND METHODS

### Ethics statement

This study was conducted according to the Declaration of Helsinki, and was approved by the institutional review boards of the National Health Research Institutes and participating hospitals.

### Participants

The Healthy Aging Longitudinal Study in Taiwan (HALST) is an ongoing community-based prospective cohort study comprising 5664 adults aged 55 years and older living in Taiwan [62]. Baseline examinations, consisting of a home visit and clinical examination, were conducted between 2009 and 2013. After providing informed consent, each participant received standardized assessments of sociodemographic status, health status, physical function, geriatric conditions, and clinical and laboratory examinations. A total of 5329 individuals who underwent a complete grip strength assessment and a laboratory test for serum uric acid were included in this study.

### Uric acid assessment

During baseline examinations, venous blood samples were collected for laboratory analysis. Uric acid was determined enzymatically using the ADVIA^®^ 1800 Chemistry System (Siemens AG, Munich, Germany). Blinded duplicate measurements were randomly performed for 5% of the specimens. The intra-assay coefficient of variation (CV) was 1.34%.

### Grip strength assessment

Grip strength (kg) was measured using a standard calibrated hand dynamometer (North Coast Medical Inc, Gilroy, CA, USA) [63]. During the assessment, the participant sat on a chair with their upper arm naturally sagging and the elbow at 90°. Each hand performed three grips on the dynamometer under the instruction and supervision of trained fieldworkers. The highest recorded grip strength was used for analysis. The grip strength lower than the median of the sex-specific distribution (≤22 kg in women; ≤36 kg in men) were considered low [63].

### Measurement of clinical indicators of biological aging

Routine clinical examination and laboratory analysis of fasting blood and morning urine sample was performed during baseline examinations in the HALST [62]. We analyzed the data of 32 clinical biomarkers of physiological systems (i.e., the liver, metabolic system, cardiovascular system, lungs, kidneys, immune system, and hematologic system) that have been applied in the assessment of biological aging in humans: 1) Liver: blood levels of alanine aminotransferase (ALT), aspartate aminotransferase (AST), albumin, ratio of albumin to globulin (A/G ratio), gamma glutamyltransferase (GGT), and platelet count; 2) Metabolic system: blood levels of hemoglobin A1C (HbA1C), glucose, insulin, triglycerides, total cholesterol, high-density lipoprotein (HDL) cholesterol, and low-density lipoprotein (LDL) cholesterol; 3) Cardiovascular system: systolic blood pressure (SBP), diastolic blood pressure (DBP), pulse rate, and ratio of urinary albumin to urinary creatinine (ACR); 4) Lungs: peak expiratory flow (PEF); 5) Kidneys: blood level of creatinine (Cr) and blood urea nitrogen (BUN); 6) Immune system: blood levels of high-sensitivity C-reactive protein (hs-CRP), interleukin-6 (IL-6), tumor necrosis factor receptor 1 (TNFR1), white blood cell (WBC) count, absolute lymphocyte number, absolute monocyte number, and absolute granulocyte number; 7) Hematologic system: red blood cell (RBC) count, hemoglobin, hematocrit, mean corpuscular volume (MCV), and red blood cell distribution width (RDW) [64–67]. Forced expiratory volume and blood levels of alkaline phosphatase were not measured in HALST; we analyzed PEF and GGT instead. Because uric acid levels are closely related to the vascular system, we also analyzed ACR, an indicator of vascular and kidney damage [68].

Blood levels of ALT, AST, albumin, globulin, and GGT were measured using the ADVIA^®^ 1800 Chemistry System (Siemens AG, Munich, Germany), with CVs of 5.83%, 5.84%, 1.17%, 2.19%, and 4.90%, respectively. Platelet count was measured using an automated hematology analyzer (XE-2100; Sysmex, Kobe, Japan), and the coefficient of variation was 2.97%. The HbA_1c_ level was measured through ion-exchange high-performance liquid chromatography by using the Variant II Turbo 2.0 System (Bio-Rad Laboratories, CA, USA), with an intra-assay CV of 1.04%. Blood levels of glucose, triglycerides, total cholesterol, HDL cholesterol, and LDL cholesterol were determined enzymatically using the ADVIA^®^ 1800 Chemistry System (Siemens AG, Munich, Germany), with CVs of 1.18%, 3.57%, 1.20%, 2.38%, and 1.94%, respectively. The blood level of insulin was measured using an automated chemiluminescence assay (ADVIA Centaur, Siemens Medical Solutions, Fernwald, Germany), with a CV of 8.52%. By using an Omron HEM-907 automated BP device (Omron Healthcare Co., Kyoto, Japan), seated resting blood pressure was measured in the right arm three times, with an interval of 1 min between measurements. The average of the final two readings was used for analysis. The levels of albumin and creatinine in spot urine specimens were determined through polyethylene-glycol-enhanced immunoturbidimetry and the Jaffe method by using the ADVIA^®^ 1800 Chemistry System, with CVs of 17.29% and 3.86%, respectively. After receiving instructions from trained fieldworkers and under their supervision, HALST participants underwent PEF measurement three times by using a Mini-Wright peak flow meter (Mini-Wright Standard, Clement Clarke International, Harlow, Essex, UK) and disposable mouthpieces according to a standardized protocol. The maximum value was used for analysis.

The blood level of creatinine and BUN were determined using the ADVIA^®^ 1800 Chemistry System (Siemens AG), with CVs of 3.13% and 3.58%, respectively. The hs-CRP was measured using the latex-enhanced immunoturbidimetric assay with the ADVIA 1800 Chemistry system; the intra-assay CV was 5.61%, and the lowest detectable concentration was 0.12 mg/L. IL-6 was measured using a highly sensitive enzyme-linked immunosorbent assay (Human IL-6 Quantikine HS ELISA Kit, R&D Systems Inc.); the intra-assay CV was 16.64%, and the minimum detectable level was 0.039 pg/mL. TNFR1 was measured using an enzyme-linked immunosorbent assay (Human sTNF-R1 ELISA kit, R&D Systems Inc.); the intra-assay CV was 9.64%, and the lowest detectable concentration was 0.77 pg/mL. Complete blood count was measured using an automated hematology analyzer (XE-2100, Sysmex, Kobe, Japan). The coefficients of variation were as follows: WBC count, 3.95%; lymphocytes, 2.97%; monocytes, 9.04%; granulocytes, 1.83%; RBC count, 0.75%; hemoglobin, 0.81%; hematocrit, 0.69%; MCV, 0.30%; and RDW, 0.57%.

### Measurement of potential confounders

The potential confounders in this study include age, sex, marital status, behavioral characteristics (i.e., smoking [never, ever, and current], alcohol drinking [never, ever, and current], and betel nut chewing [never, ever, and current]), body mass index (BMI), comorbidities (i.e., hypertension, diabetes mellitus, stroke, cardiovascular disease, arthritis, chronic kidney disease, cancer, and lung disease), and use of uric acid–lowering drugs (see Supplementary Materials and Methods for more detail).

### All-cause mortality

The primary outcome was all-cause mortality. Participants were linked to data on clinical death events from the Bureau of National Health Insurance of Taiwan death registry to ascertain any death events that occurred during the follow-up period. We followed up each participant from the date of baseline assessment (index date) until death or the end of the study (May 2020), whichever occurred first.

### Statistical analysis

Descriptive statistics were used to examine the distribution of subject characteristics. Normally distributed continuous variables are presented as mean (standard deviation [SD]), and nonnormally distributed data are presented as median (interquartile range). Nonnormally distributed data were normalized using the natural log transformation. The differences between continuous variables were analyzed using one-way ANOVA. The differences between categorical variables were analyzed using a chi-square test.

Cox proportional-hazards regression models were used to analyze the relationship between serum uric acid and mortality risk, adjusted for confounders. We first examined the effect of grip strength on the relationship between uric acid and mortality risk. We added the interaction term between uric acid levels and grip strength (either as a categorical or continuous variable), in addition to their main effects, into the model and tested its significance. Because it was significant, the study population was stratified by grip strength. We then nonparametrically examined the relation between serum uric acid and mortality risk separately in participants with and without low grip strength using restricted cubic splines with adjustment for confounders [69]. Four knots were placed at the 5th, 35th, 65th, and 95th percentiles. Nonlinearity was tested using the likelihood-ratio test, wherein the model with only the linear term was compared with the model with the linear and the cubic spline terms. Finally, we divided the study population into six groups based on grip strength (high and low) and uric acid (low, medium, and high). We estimated the hazard ratios of all-cause mortality and 95% confidence interval (CI) for each group. We adjusted the impact of possible confounders by constructing models that additionally included them. To obtain parsimonious models with significantly enhanced fit, we selected variables in a stepwise manner based on the Akaike information criterion. The proportional hazards assumption was checked by including time-dependent covariates in the models. The assumptions were met (*P* >.05). In these analyses of mortality risk, follow-up time was censored at the end of the follow-up (May 2020) for those who were still alive.

We investigated the robustness of the study results through the following sensitivity analysis. We randomly split the whole cohort in half and repeated the analysis in each of the two sub-cohorts. To explore the possible influence of uric acid–lowering medication on the results, we excluded participants taking uric acid-lowering medication and repeated the analysis. In addition, to examine the possible impact of reverse causation, we excluded the individuals who died within the first year during follow-up. Finally, we conducted an analysis that takes into account the left truncation.

We explored the differences in the levels of each of the 32 clinical indicators of biological aging among older adults with different serum uric acid levels and grip strengths by using the general linear model with adjustment for the type I error of multiple comparisons. To further delineate the relationship between uric acid and inflammatory markers of biological aging, we regressed the levels of blood hs-CRP, IL-6, and TNFR1 against serum uric acid levels and other potential confounders through restricted cubic spline regression analyses.

In this study, a *P*-value of <.05 was considered statistically significant. We used SAS version 9.4 software (SAS Institute Inc., Cary, NC, USA) for analysis.

## Supplementary Material

Supplementary Materials and Methods

Supplementary Figures

Supplementary Tables
