# Supplementary material for: Differential relationship of uric acid to mortality and clinical biomarkers of aging according to grip strength in older adults: a cohort study
Source: Aging (Albany NY). 2021 Apr 4;13(7):10555–83. doi: 10.18632/aging.202820 (PMC8064196; doi:10.18632/aging.202820)
Supplement: Supplementary Materials and Methods [file aging-13-202820-s001.pdf]

SUPPLEMENTARY MATERIALS AND METHODS

Measurement of potential confounders

BMI was calculated as body weight (kg) divided by the square of the height (m^2) and was categorized according to the WHO criteria recommended for Asians (overweight: $23 \leq BMI < 27.5$; obesity: $BMI \geq 27.5$) [1]. Comorbidities included hypertension, diabetes mellitus, stroke, cardiovascular disease, arthritis, chronic kidney disease, cancer and lung disease. The presence of hypertension was based on self-report, antihypertensive drug treatment or an average blood pressure of ≥140/90 mmHg [2]. Diabetes mellitus was defined by self-report, medication use, fasting blood glucose of 126 mg/dL or higher or HbA1c of ≥6.5% [3]. Chronic kidney disease was defined as an estimated glomerular filtration rate of <60 $mL \cdot min^{-1} (1.73 m^2)^{-1}$ [4]. Other diseases were assessed by referring to the self-reported physician's diagnosis. Use of uric acid lowering drug was determined by assessing whether individuals were taking medicine with contents of any one of below: Allopurinol, Febuxostat, Probenecid, Sulfinpyrazone, Benzbromarone or Rasburicase.

REFERENCES

1. WHO Expert Consultation. Appropriate body-mass index for Asian populations and its implications for policy and intervention strategies. Lancet. 2004; 363:157–63. https://doi.org/10.1016/s0140-6736(03)15268-3 PMID:14726171

2. Chobanian AV, Bakris GL, Black HR, Cushman WC, Green LA, Izzo JL Jr, Jones DW, Materson BJ, Oparil S, Wright JT Jr, Roccella EJ, and Joint National Committee on Prevention, Detection, Evaluation, and Treatment of High Blood Pressure. Seventh report of the Joint National Committee on Prevention, Detection, Evaluation, and Treatment of High Blood Pressure. Hypertension. 2003; 42:1206–52. https://doi.org/10.1161/01.hyp.0000107251.49515.c2 PMID:14656957

3. American Diabetes Association. Standards of medical care in diabetes--2014. Diabetes Care. 2014 (Suppl 1); 37:S14–80. https://doi.org/10.2337/dc14-s014 PMID:24357209

4. National Kidney Foundation. K/DOQI clinical practice guidelines for chronic kidney disease: evaluation, classification, and stratification. Am J Kidney Dis. 2002 (Suppl 1); 39:S1–266. PMID:11904577
