# Supplementary material for: Differential relationship of uric acid to mortality and clinical biomarkers of aging according to grip strength in older adults: a cohort study
Source: Aging (Albany NY). 2021 Apr 4;13(7):10555–83. doi: 10.18632/aging.202820 (PMC8064196; doi:10.18632/aging.202820)
Supplement: Supplementary Figures [file aging-13-202820-s002.pdf]

SUPPLEMENTARY FIGURES

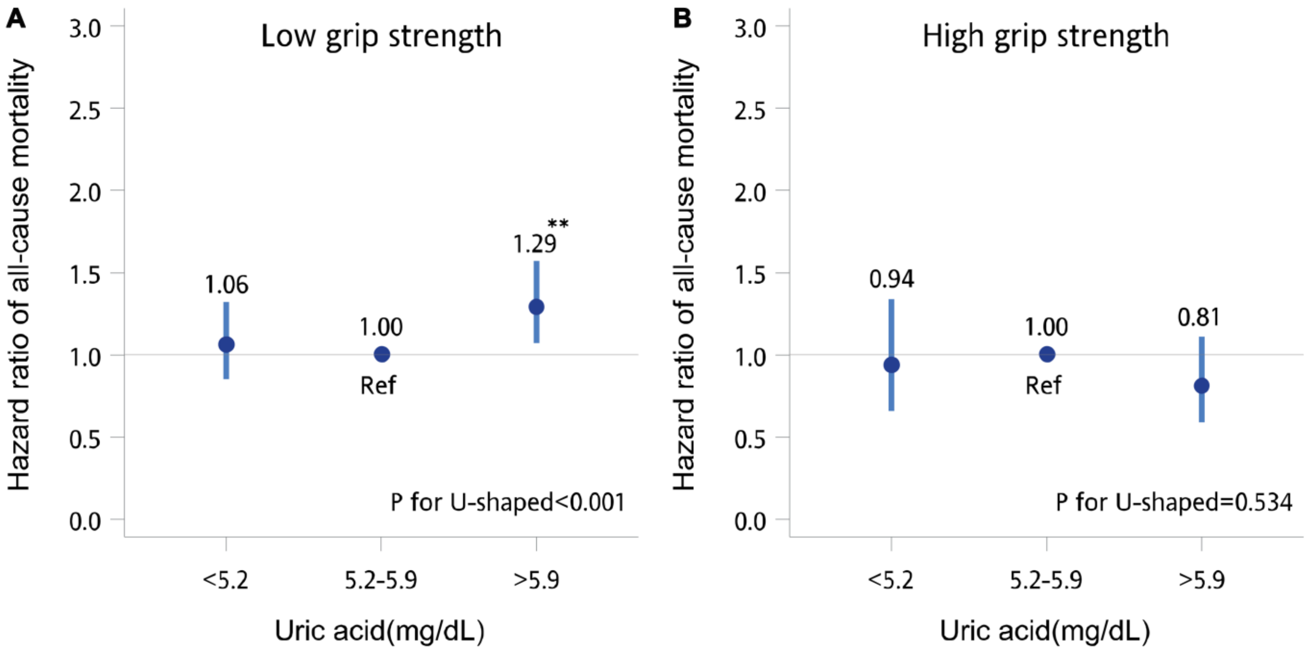

Supplementary Figure 1. Adjusted hazard ratios of all-cause mortality by serum uric acid levels in older adults with low grip strength and those with high grip strength. The adjusted hazard ratios of all-cause mortality for low (<5.2 mg/dl), medium (5.2–5.9 mg/dl) and high (≥6.0 mg/dl) serum uric acid in older adults with low grip strength (**A**) and high grip strength (**B**) are presented. The multivariate models were adjusted for age, sex, marital status, behavioral characteristics, body mass index, chronic diseases, and use of uric acid-lowering drugs. Error bars indicate 95% confidence intervals. Asterisk indicates P < .05; and double asterisk, P < .01 for comparisons with the reference group (Ref).

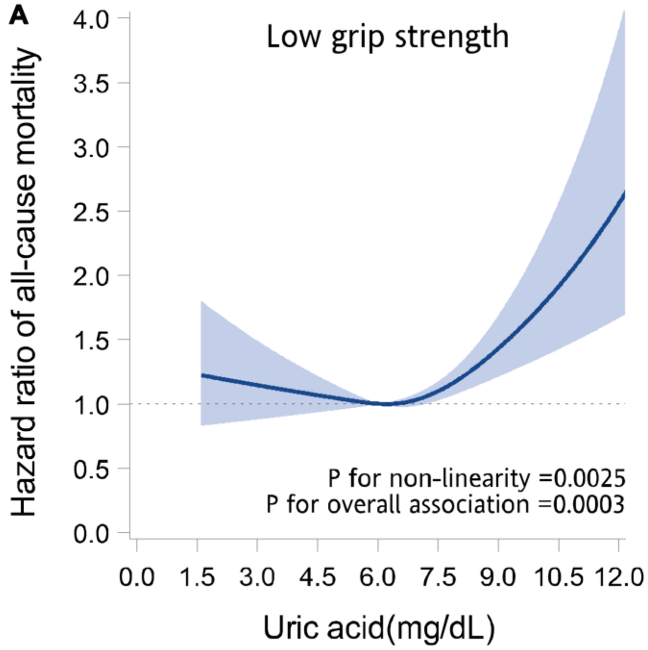

Supplementary Figure 2. Association between serum uric acid and risk of death after excluding participants taking uric acid lowering medications. The graphs demonstrated the adjusted hazard ratios for all-cause mortality by serum uric acid levels in older adults with low grip strength (**A**) and those with high grip strength (**B**). The hazard ratios (solid line) and 95% confidence intervals (band) are estimated by fitting restricted cubic spline Cox regression models in which uric acid was modeled as a continuous variable with splines having four knots placed at the 5th, 35th, 65th and 95th percentiles. Models are adjusted for age, sex, marital status, behavioral characteristics, body mass index and chronic diseases.

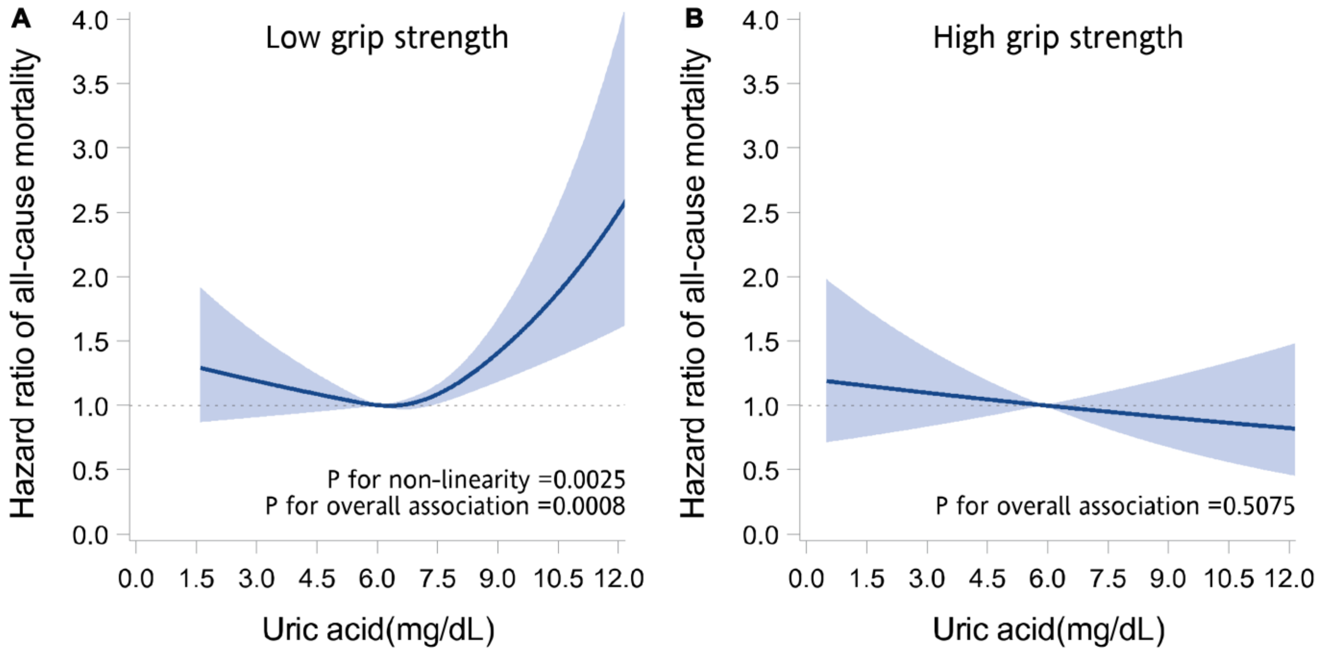

Supplementary Figure 3. Association between serum uric acid and risk of death after additionally excluding participants who died within the first year during following up. The graphs demonstrated the adjusted hazard ratios for all-cause mortality by serum uric acid levels in older adults with low grip strength (**A**) and those with high grip strength (**B**). The hazard ratios (solid line) and 95% confidence intervals (band) are estimated by fitting restricted cubic spline Cox regression models in which uric acid was modeled as a continuous variable with splines having four knots placed at the 5th, 35th, 65th and 95th percentiles. Models are adjusted for age, sex, marital status, behavioral characteristics, body mass index and chronic diseases.

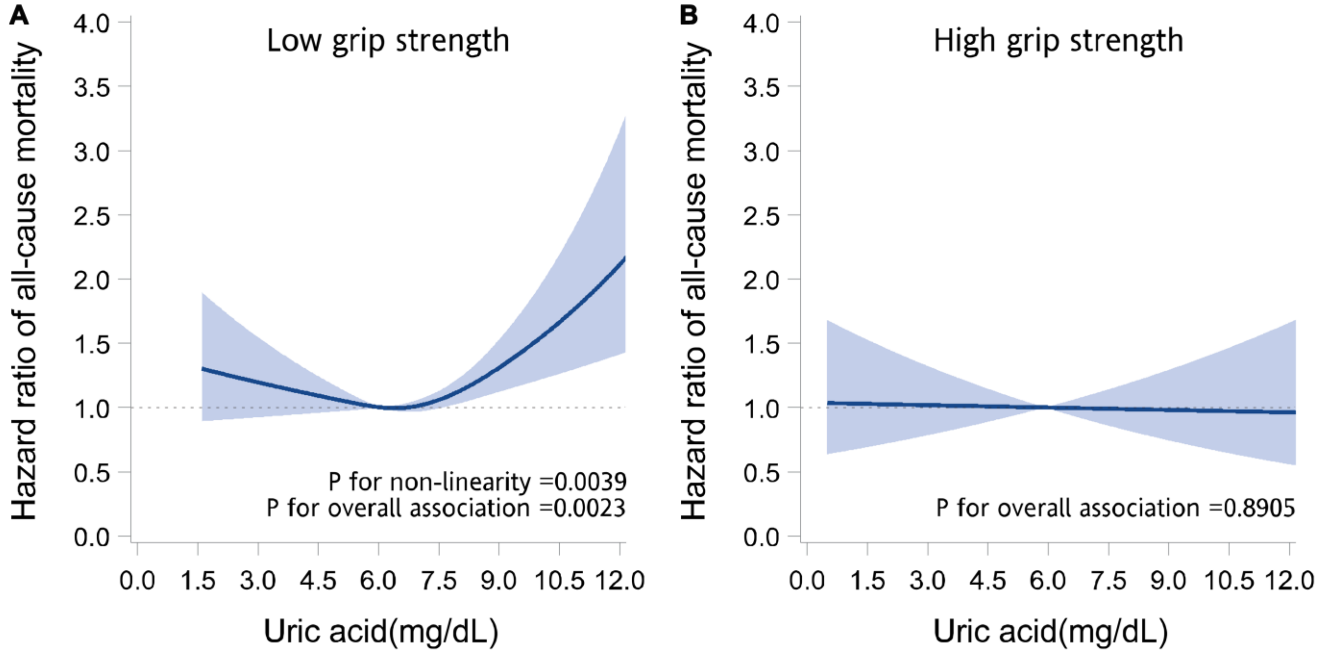

Supplementary Figure 4. Association between serum uric acid and risk of death after taking into account left truncation. The graphs demonstrated the adjusted hazard ratios for all-cause mortality by serum uric acid levels in older adults with low grip strength (**A**) and those with high grip strength (**B**). The hazard ratios (solid line) and 95% confidence intervals (band) are estimated by fitting restricted cubic spline Cox regression models in which uric acid was modeled as a continuous variable with splines having four knots placed at the 5th, 35th, 65th and 95th percentiles. Models are adjusted for age, sex, marital status, behavioral characteristics, body mass index and chronic diseases.

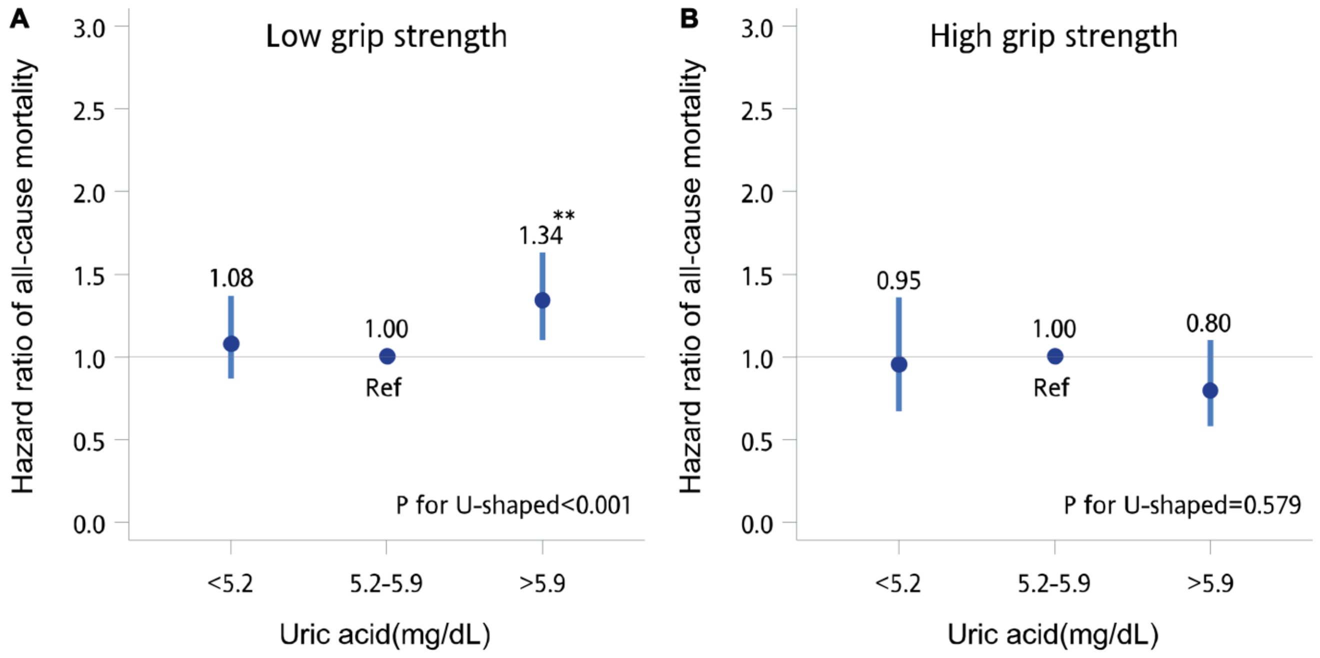

Supplementary Figure 5. Adjusted hazard ratios of all-cause mortality by serum uric acid levels in older adults with low grip strength and those with high grip strength after excluding participants taking uric acid lowering medications. The adjusted hazard ratios of all-cause mortality for low (<5.2 mg/dl), medium (5.2–5.9 mg/dl) and high (≥6.0 mg/dl) serum uric acid in older adults with low grip strength (**A**) and high grip strength (**B**) are presented. The multivariate models were adjusted for age, sex, marital status, behavioral characteristics, body mass index, and chronic diseases. Error bars indicate 95% confidence intervals. Asterisk indicates *P* < .05; and double asterisk, *P* < .01 for comparisons with the reference group (Ref).

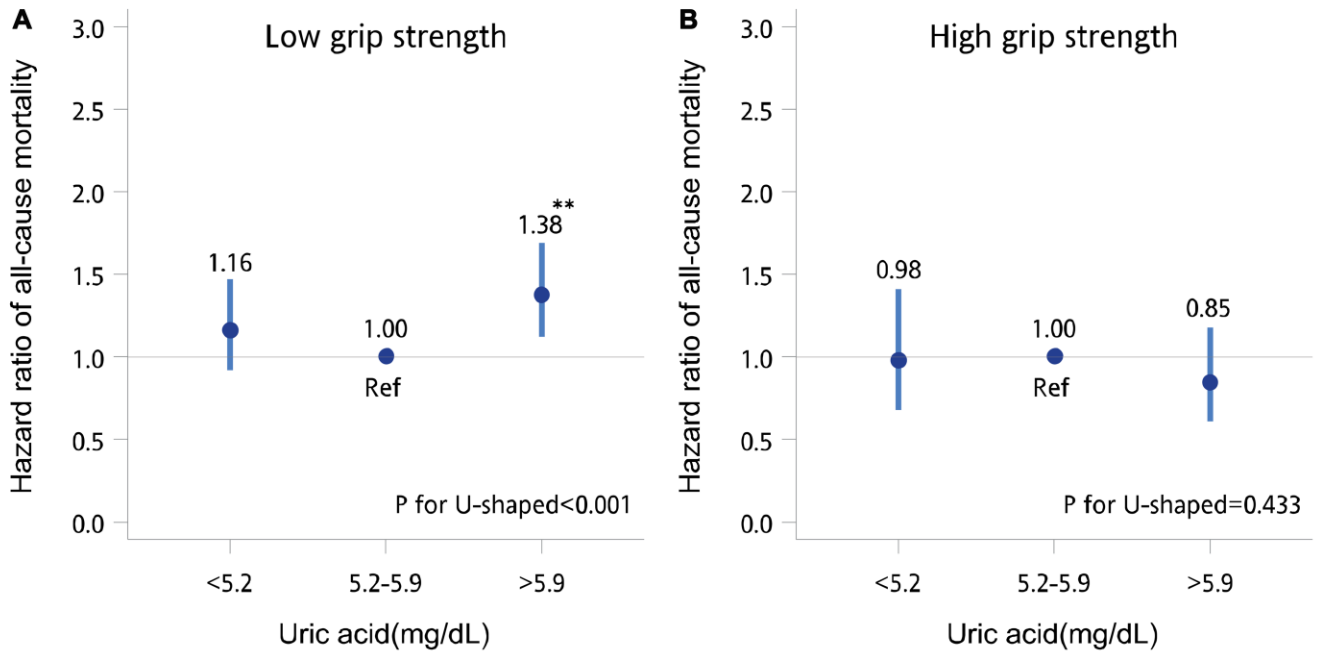

Supplementary Figure 6. Adjusted hazard ratios of all-cause mortality by serum uric acid levels in older adults with low grip strength and those with high grip strength after additionally excluding participants who died within the first year during following up. The adjusted hazard ratios of all-cause mortality for low (<5.2 mg/dl), medium (5.2–5.9 mg/dl) and high (≥6.0 mg/dl) serum uric acid in older adults with low grip strength (**A**) and high grip strength (**B**) are presented. The multivariate models were adjusted for age, sex, marital status, behavioral characteristics, body mass index, and chronic diseases. Error bars indicate 95% confidence intervals. Asterisk indicates *P* < .05; and double asterisk, *P* < .01 for comparisons with the reference group (Ref).

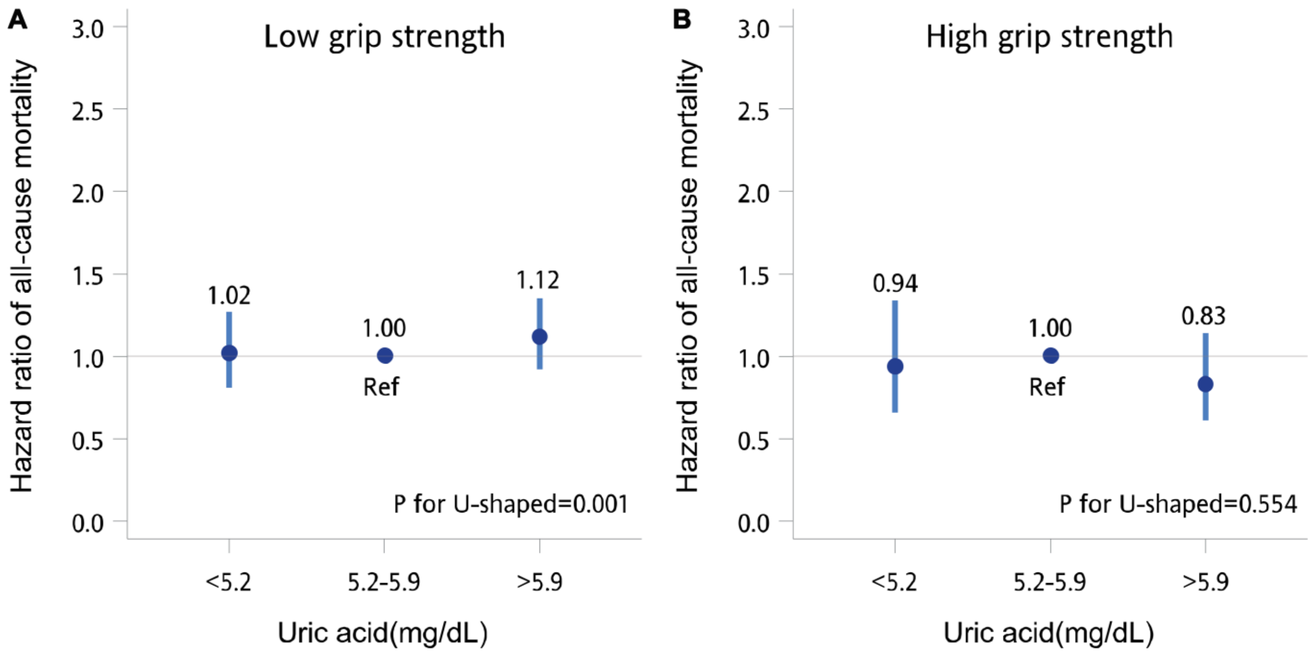

Supplementary Figure 7. Adjusted hazard ratios for all-cause mortality by serum uric acid levels in older adults with low grip strength and those with high grip strength after taking into account left truncation. The adjusted hazard ratios of all-cause mortality for low (<5.2 mg/dl), medium (5.2–5.9 mg/dl) and high (≥6.0 mg/dl) serum uric acid in older adults with low grip strength (**A**) and high grip strength (**B**) are presented. The multivariate models were adjusted for age, sex, marital status, behavioral characteristics, body mass index, chronic diseases, and use of uric acid–lowering drugs. Error bars indicate 95% confidence intervals. Asterisk indicates *P* < .05; and double asterisk, *P* < .01 for comparisons with the reference group (Ref).

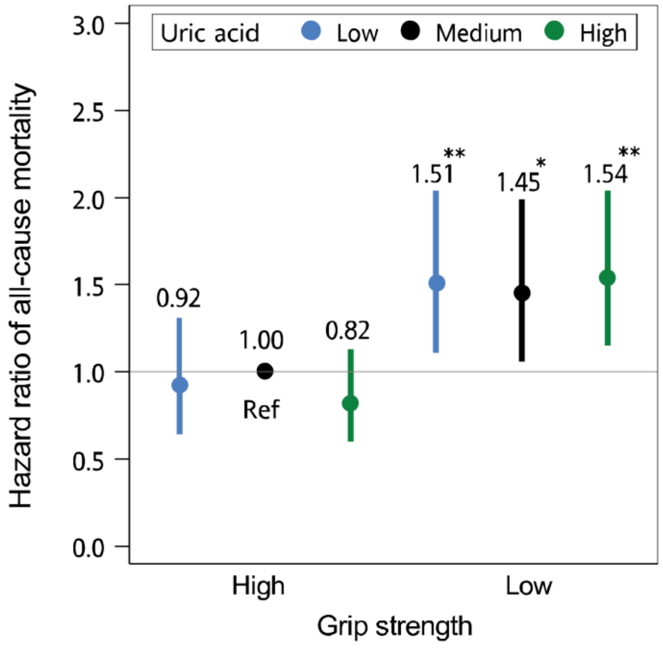

Supplementary Figure 8. Joint effect of grip strength and serum uric acid levels on risk of death after excluding participants taking uric acid lowering medications. The hazard ratios for all-cause mortality according to grip strength (high [>22kg in women, >36kg in men] and low [≤22kg in women, ≤36kg in men]) and serum uric acid levels (low [<5.2 mg/dl], medium [5.2–5.9 mg/dl] and high [≥6.0 mg/dl]) are presented. The multivariate models are adjusted for age, sex, marital status, behavioral characteristics, body mass index and chronic diseases. Error bars indicate 95% confidence intervals. Asterisk indicates *P* <.05; and double asterisk, *P* <.01 for comparisons with the reference group (Ref).

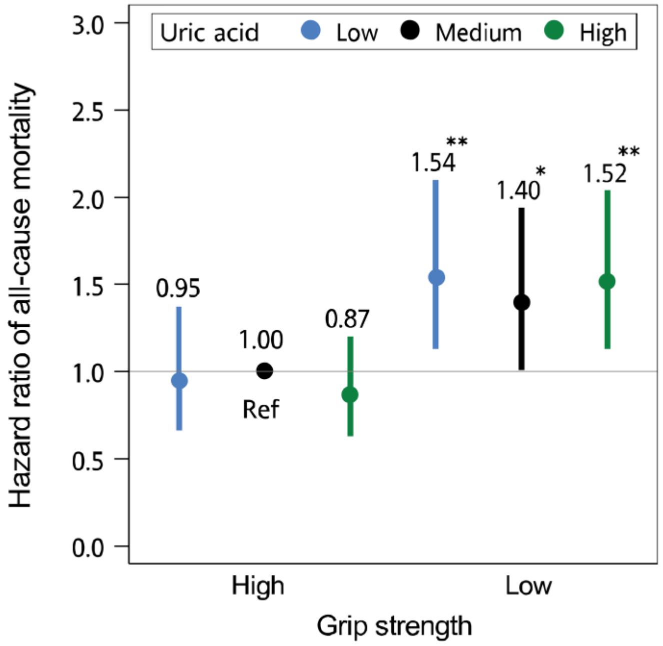

Supplementary Figure 9. Joint effect of grip strength and serum uric acid levels on risk of death after additionally excluding participants who died within the first year during following up. The hazard ratios for all-cause mortality according to grip strength (high [>22kg in women, >36kg in men] and low [≤22kg in women, ≤36kg in men]) and serum uric acid levels (low [<5.2 mg/dl], medium [5.2–5.9 mg/dl] and high [≥6.0 mg/dl]) are presented. The multivariate models are adjusted for age, sex, marital status, behavioral characteristics, body mass index and chronic diseases. Error bars indicate 95% confidence intervals. Asterisk indicates *P* < .05; and double asterisk, *P* < .01 for comparisons with the reference group (Ref).

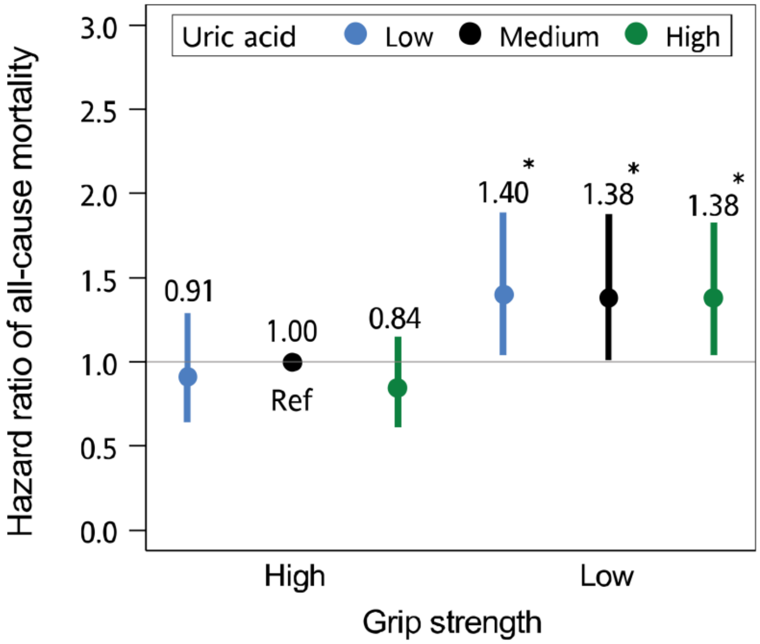

Supplementary Figure 10. Joint effect of grip strength and serum uric acid levels on risk of death after taking into account left truncation. The hazard ratios for all-cause mortality according to grip strength (high [>22 kg in women, >36 kg in men] and low [≤22kg in women, ≤36 kg in men]) and serum uric acid levels (low [<5.2 mg/dl], medium [5.2–5.9 mg/dl] and high [≥6.0 mg/dl]) are presented. The multivariate models are adjusted for age, sex, marital status, behavioral characteristics, body mass index and chronic diseases. Error bars indicate 95% confidence intervals. Asterisk indicates *P* < .05; and double asterisk, *P* < .01 for comparisons with the reference group (Ref).
