# Supplementary material for: Differential relationship of uric acid to mortality and clinical biomarkers of aging according to grip strength in older adults: a cohort study
Source: Aging (Albany NY). 2021 Apr 4;13(7):10555–83. doi: 10.18632/aging.202820 (PMC8064196; doi:10.18632/aging.202820)
Supplement: Supplementary Tables [file aging-13-202820-s003.pdf]

SUPPLEMENTARY TABLES

Supplementary Table 1. Clinical Indicators of biological aging according to grip strength levels at baseline.

Indicators	All (*n* = 5329)	Grip strength[a] Low (*n* = 2732)	High (*n* = 2597)	*P*[b]
Liver				
Aspartate aminotransferase, U/L	26.0 (22.0–31.0)	26.0 (22.0–32.0)	26.0 (23.0–30.0)	.001
Alanine aminotransferase, U/L	22.0 (18.0–30.0)	22.0 (17.0–29.0)	23.0 (19.0–30.0)	<.001
Albumin, g/dL	4.4 (4.2–4.5)	4.4 (4.2–4.5)	4.4 (4.3–4.6)	<.001
Albumin to globulin ratio	1.5 (1.4–1.7)	1.5 (1.4–1.6)	1.6 (1.4–1.7)	<.001
Gamma glutamyltransferase, U/L	23.0 (16.0–35.0)	23.0 (17.0–35.0)	22.0 (16.0–34.0)	.002
Platelet count, 10^3/ul, mean (SD)	223.9 (60.1)	223.0 (64.5)	224.9 (55.1)	.266
Metabolic system				
Hemoglobin A1C, %	6.0 (5.7–6.4)	6.0 (5.7–6.5)	5.9 (5.6–6.3)	<.001
Glucose, mg/dL	103.0 (95.0–116.0)	103.0 (95.0–118.0)	103.0 (96.0–114.0)	.006
Insulin, uIU/mL	5.6 (3.7–8.4)	5.5 (3.6–8.3)	5.7 (3.9–8.5)	.008
Triglycerides, mg/dL	104.0 (75.0–148.0)	105.0 (75.0–148.0)	104.0 (75.5–148.0)	.539
Total cholesterol, mg/dL, mean (SD)	194.7 (36.9)	192.4 (37.5)	197.0 (36.0)	<.001
High-density lipoprotein cholesterol, mg/dL	50.0 (43.0–60.0)	50.0 (43.0–60.8)	51.0 (43.0–60.0)	.500
Low-density lipoprotein cholesterol, mg/dL, mean (SD)	118.0 (33.1)	115.7 (32.9)	120.5 (33.1)	<.001
Immune system				
High-sensitivity C-reactive protein, mg/dL	0.07 (0.02–0.21)	0.08 (0.03–0.26)	0.06 (0.02–0.17)	<.001
Interleukin-6, pg/mL	1.41 (0.94–2.24)	1.62 (1.07–2.57)	1.24 (0.85–1.89)	<.001
Tumor necrosis factor receptor 1, pg/mL	1169.8 (934.3–1449.1)	1300.6 (1015.7–1677.0)	1067.6 (877.0–1323.0)	<.001
White blood cell count, 10^9/L, mean (SD)	5.8 (1.6)	5.9 (1.7)	5..7 (1.6)	<.001
Lymphocytes absolute number, 10^9/L	1.7 (1.4–2.1)	1.7 (1.3–2.1)	1.8 (1.5–2.2)	<.001
Monocytes absolute number, 10^9/L	0.32 (0.26–0.41)	0.34 (0.27–0.43)	0.31 (0.25–0.39)	<.001
Granulocyte absolute number, 10^9/L	3.2 (2.6–4.0)	3.4 (2.7–4.2)	3.1 (2.5–3.9)	<.001
Hematologic system				
Red blood cell count, 10^{12}/L, mean (SD)	4.6 (0.5)	4.5 (0.6)	4.7 (0.5)	<.001
Hemoglobin, g/dL, mean (SD)	13.6 (1.5)	13.4 (1.5)	13.9 (1.4)	<.001
Hematocrit, %, mean (SD)	41.1 (3.9)	40.4 (4.1)	41.8 (3.6)	<.001
Mean corpuscular volume, fL	91.2 (88.3–94.0)	91.3 (88.3–94.3)	91.0 (88.3–93.7)	.070
Red blood cell distribution width, %	13.2 (12.8–13.7)	13.3 (12.9–13.9)	13.1 (12.7–13.6)	<.001
Cardiovascular system				
Systolic blood pressure, mmHg, mean (SD)	128.6 (18.8)	130.2 (19.6)	126.9 (17.8)	<.001

Diastolic blood pressure, mmHg, mean (SD)	70.6 (10.8)	69.4 (10.9)	71.8 (10.7)	<.001
Pulse rate, beats/min, mean (SD)	68.6 (10.3)	68.9 (10.7)	68.2 (9.8)	.029
Urinary albumin to creatinine ratio, mg/g	9.5 (4.6–27.8)	11.9 (5.4–41.1)	7.6 (3.9–19.4)	<.001
Lung				
Peak expiratory flow, l/min, mean (SD)	380.7 (1.7)	342.2 (119.7)	420.3 (116.2)	<.001
Kidney				
Creatinine, mg/dL	0.85 (0.70–1.02)	0.86 (0.71–1.05)	0.83 (0.69–0.99)	<.001
Blood urea nitrogen, mg/dL	16.0 (13.4–19.2)	16.5 (13.6–20.4)	15.5 (13.2–18.4)	<.001

Note: Data are median (interquartile range) unless otherwise specified.

[a]Low grip strength: women ≤ 22 kg, men ≤ 36 kg.

[b]Analyzed using one-way analysis of variance. (Non-normally distributed data were first normalized.)

Supplementary Table 2. Hazard ratios for all-cause mortality according to grip strength and serum uric acid levels.

Variables	Hazard Ratio	95% CI	*P*
Levels of grip strength and uric acid[a]			
High grip strength & low uric acid	0.91	0.64 to 1.29	.595
High grip strength & medium uric acid	1.00	(Reference)	
High grip strength & high uric acid	0.83	0.61 to 1.13	.240
Low grip strength & low uric acid	1.52	1.13 to 2.05	<.001
Low grip strength & medium uric acid	1.49	1.09 to 2.03	.012
Low grip strength & high uric acid	1.52	1.15 to 2.02	<.001
Age	1.08	1.07 to 1.09	<.001
Sex			
Women	1.00	(Reference)	
Men	1.90	1.65 to 2.19	<.001
Marital status			
Not married	1.00	(Reference)	
Married	0.74	0.64 to 0.85	<.001
Body mass index			
Underweight	1.52	1.13 to 2.05	.006
Normal	1.00	(Reference)	
Overweight	0.78	0.68 to 0.9	<.001
Obese	0.83	0.69 to 1.01	.059
Diabetes mellitus	1.48	1.3 to 1.68	<.001
Cardiovascular disease	1.14	0.99 to 1.31	.060
Stroke	1.69	1.39 to 2.06	<.001
Lung disease	1.47	1.14 to 1.91	<.001
Cancer	1.51	1.2 to 1.89	<.001
Chronic kidney disease	1.59	1.37 to 1.83	<.001
-2 Log likelihood		16134.8	
AIC		16168.9	

[a]Categorized according to grip strength (high [>22kg in women, >36kg in men] and low [≤22kg in women, ≤36kg in men]) and serum uric acid levels (low [<5.2 mg/dl], medium [5.2–5.9 mg/dl] and high [≥6.0 mg/dl]).

Supplementary Table 3. Hazard ratios for all-cause mortality according to grip strength and serum uric acid levels after excluding participants taking uric acid lowering medications.

Variables	Hazard Ratio	95% CI	*P*
Levels of grip strength and uric acid[a]			
High grip strength & low uric acid	0.92	0.64 to 1.31	.636
High grip strength & medium uric acid	1.00	(Reference)	
High grip strength & high uric acid	0.82	0.60 to 1.13	.233
Low grip strength & low uric acid	1.51	1.11 to 2.04	<.001
Low grip strength & medium uric acid	1.45	1.06 to 1.99	.020
Low grip strength & high uric acid	1.54	1.15 to 2.04	<.001
Age	1.08	1.07 to 1.09	<.001
Sex			
Women	1.00	(Reference)	
Men	1.94	1.68 to 2.25	<.001
Marital status			
Not married	1.00	(Reference)	
Married	0.73	0.63 to 0.85	<.001
Body mass index			
Underweight	1.51	1.12 to 2.03	<.001
Normal	1.00	(Reference)	
Overweight	0.76	0.66 to 0.88	<.001
Obese	0.81	0.67 to 0.98	.033
Diabetes mellitus	1.51	1.32 to 1.72	<.001
Cardiovascular disease	1.13	0.98 to 1.31	.084
Stroke	1.68	1.37 to 2.07	<.001
Lung disease	1.45	1.11 to 1.89	<.001
Cancer	1.52	1.2 to 1.91	<.001
Chronic kidney disease	1.56	1.34 to 1.81	<.001
-2 Log likelihood		15294.8	
AIC		15328.8	

[a]Categorized according to grip strength (high [>22kg in women, >36kg in men] and low [≤22kg in women, ≤36kg in men]) and serum uric acid levels (low [<5.2 mg/dl], medium [5.2–5.9 mg/dl] and high [≥6.0 mg/dl]).

Supplementary Table 4. Hazard ratios for all-cause mortality according to grip strength and serum uric acid levels after additionally excluding participants who died within the first year during following up.

Variables	Hazard Ratio	95% CI	*P*
Levels of grip strength and uric acid[a]			
High grip strength & low uric acid	0.95	0.66 to 1.37	.786
High grip strength & medium uric acid	1.00	(Reference)	
High grip strength & high uric acid	0.87	0.63 to 1.20	.398
Low grip strength & low uric acid	1.54	1.13 to 2.10	<.001
Low grip strength & medium uric acid	1.40	1.01 to 1.94	.043
Low grip strength & high uric acid	1.52	1.13 to 2.04	<.001
Age	1.09	1.08 to 1.1	<.001
Sex			
Women	1.00	(Reference)	
Men	1.92	1.65 to 2.22	<.001
Marital status			
Not married	1.00	(Reference)	
Married	0.74	0.63 to 0.86	<.001
Body mass index			
Underweight	1.53	1.13 to 2.08	<.001
Normal	1.00	(Reference)	
Overweight	0.76	0.65 to 0.88	<.001
Obese	0.80	0.66 to 0.98	.033
Diabetes mellitus	1.51	1.32 to 1.73	<.001
Cardiovascular disease	1.12	0.97 to 1.3	.127
Stroke	1.71	1.38 to 2.11	<.001
Lung disease	1.47	1.12 to 1.93	<.001
Cancer	1.49	1.17 to 1.89	<.001
Chronic kidney disease	1.55	1.33 to 1.81	<.001
-2 Log likelihood		14631.1	
AIC		14665.1	

[a]Categorized according to grip strength (high [>22kg in women, >36kg in men] and low [≤22kg in women, ≤36kg in men]) and serum uric acid levels (low [<5.2 mg/dl], medium [5.2–5.9 mg/dl] and high [≥6.0 mg/dl]).

Supplementary Table 5. Hazard ratios for all-cause mortality according to grip strength and serum uric acid levels after taking into account left truncation.

Variables	Hazard Ratio	95% CI	*P*
Levels of grip strength and uric acid[a]			
High grip strength & low uric acid	0.91	0.64 to 1.29	.584
High grip strength & medium uric acid	1.00	(Reference)	
High grip strength & high uric acid	0.84	0.61 to 1.15	.274
Low grip strength & low uric acid	1.40	1.04 to 1.89	.043
Low grip strength & medium uric acid	1.38	1.01 to 1.88	.044
Low grip strength & high uric acid	1.38	1.04 to 1.83	.025
Sex			
Women	1.00	(Reference)	
Men	1.81	1.57 to 2.09	<.001
Marital status			
Not married	1.00	(Reference)	
Married	0.80	0.70 to 0.93	.003
Body mass index			
Underweight	1.48	1.10 to 2.00	.003
Normal	1.00	(Reference)	
Overweight	0.81	0.70 to 0.93	.004
Obese	0.78	0.70 to 1.05	.150
Diabetes mellitus	1.50	1.32 to 1.71	<.001
Cardiovascular disease	1.09	0.95 to 1.25	.214
Stroke	1.66	1.36 to 2.03	<.001
Lung disease	1.46	1.16 to 1.83	.001
Cancer	1.46	1.16 to 1.83	.001
Chronic kidney disease	1.47	1.27 to 1.69	<.001
-2 Log likelihood		13832.2	
AIC		13864.2	

[a]Categorized according to grip strength (high [>22kg in women, >36kg in men] and low [≤22kg in women, ≤36kg in men]) and serum uric acid levels (low [<5.2 mg/dl], medium [5.2–5.9 mg/dl] and high [≥6.0 mg/dl]).
